# The heat shock protein-90 co-chaperone, Cyclophilin 40, promotes ALK-positive, anaplastic large cell lymphoma viability and its expression is regulated by the NPM-ALK oncoprotein

**DOI:** 10.1186/1471-2407-12-229

**Published:** 2012-06-08

**Authors:** Joel D Pearson, Zubair Mohammed, Julinor T C Bacani, Raymond Lai, Robert J Ingham

**Affiliations:** 1 Department of Medical Microbiology and Immunology, University of Alberta, Edmonton, T6G 2E1, Canada; 2 Department of Laboratory Medicine and Pathology, University of Alberta, Edmonton, T6G 2B7, Canada

## Abstract

**Background:**

Anaplastic lymphoma kinase-positive, anaplastic large cell lymphoma (ALK+ ALCL) is a T cell lymphoma defined by the presence of chromosomal translocations involving the *ALK* tyrosine kinase gene. These translocations generate fusion proteins (e.g. NPM-ALK) with constitutive tyrosine kinase activity, which activate numerous signalling pathways important for ALK+ ALCL pathogenesis. The molecular chaperone heat shock protein-90 (Hsp90) plays a critical role in allowing NPM-ALK and other signalling proteins to function in this lymphoma. Co-chaperone proteins are important for helping Hsp90 fold proteins and for directing Hsp90 to specific clients; however the importance of co-chaperone proteins in ALK+ ALCL has not been investigated. Our preliminary findings suggested that expression of the immunophilin co-chaperone, Cyclophilin 40 (Cyp40), is up-regulated in ALK+ ALCL by JunB, a transcription factor activated by NPM-ALK signalling. In this study we examined the regulation of the immunophilin family of co-chaperones by NPM-ALK and JunB, and investigated whether the immunophilin co-chaperones promote the viability of ALK+ ALCL cell lines.

**Methods:**

NPM-ALK and JunB were knocked-down in ALK+ ALCL cell lines with siRNA, and the effect on the expression of the three immunophilin co-chaperones: Cyp40, FK506-binding protein (FKBP) 51, and FKBP52 examined. Furthermore, the effect of knock-down of the immunophilin co-chaperones, either individually or in combination, on the viability of ALK+ ALCL cell lines and NPM-ALK levels and activity was also examined.

**Results:**

We found that NPM-ALK promoted the transcription of *Cyp40* and *FKBP52*, but only *Cyp40* transcription was promoted by JunB. We also observed reduced viability of ALK+ ALCL cell lines treated with Cyp40 siRNA, but not with siRNAs directed against FKBP52 or FKBP51. Finally, we demonstrate that the decrease in the viability of ALK+ ALCL cell lines treated with Cyp40 siRNA does not appear to be due to a decrease in NPM-ALK levels or the ability of this oncoprotein to signal.

**Conclusions:**

This is the first study demonstrating that the expression of immunophilin family co-chaperones is promoted by an oncogenic tyrosine kinase. Moreover, this is the first report establishing an important role for Cyp40 in lymphoma.

## Background

Anaplastic lymphoma kinase-positive, anaplastic large cell lymphoma (ALK+ ALCL) is an aggressive non-Hodgkin lymphoma of T/null cell immunophenotype [1-3]. This lymphoma primarily presents in children, adolescents, and young adults where it accounts for 10–20% of childhood non-Hodgkin lymphomas [1]. ALK+ ALCL is characterized by the presence of chromosomal translocations involving the *ALK* gene, which encodes for a receptor tyrosine kinase belonging to the insulin receptor super-family. These translocations result in the expression of ALK fusion proteins that are critical for the pathogenesis of ALK+ ALCL [2,3]. Moreover, ALK fusion proteins have been implicated in the pathogenesis of a subset of non-small cell lung carcinomas (ALK+ NSCLC) [4-7] and inflammatory myofibroblastic tumours (ALK+ IMT) [8-10]. In ALK+ ALCL several different *ALK* translocations have been described [2,3]; however, the most common (~80%) is the t(2;5)(p23;q35) translocation involving the *nucleophosmin (NPM)* gene which generates the NPM-ALK oncogene [1-3].

NPM-ALK consists of the N-terminal region of NPM and the C-terminal kinase and intracellular domains of ALK [11,12]. The NPM portion of this fusion protein possesses a dimerization domain required for the tyrosine kinase activity and transforming ability of NPM-ALK [13,14]. The activity of the NPM-ALK oncoprotein is also critically dependent on the molecular chaperone, heat shock protein-90 (Hsp90) [15-18]. Hsp90 is a ubiquitously expressed protein that assists in the proper folding and activity of numerous cellular proteins [19,20]. Hsp90 promotes the stability of NPM-ALK [15-18], as treatment of cell lines with the Hsp90 inhibitor, 17-Allylamino-Demethoxygeldanamycin (17-AAG), resulted in the proteasomal degradation of NPM-ALK [17]. The treatment of ALK+ ALCL cell lines with 17-AAG resulted in cell cycle arrest and the induction of apoptosis [15,18]; however, these effects are likely due to more than just decreased NPM-ALK levels. Hsp90 inhibition also decreased levels of the pro-survival serine/threonine kinase Akt, the cell cycle-associated proteins cyclin D1, cyclin-dependent kinase 4 (cdk4), and cdk6, as well as several other proteins in ALK+ ALCL [15,18,21]. The treatment of ALK+ ALCL cell lines with 17-AAG resulted in decreased phosphorylation of the serine/threonine kinase Erk without affecting Erk levels [15]. Moreover, the treatment of ALK+ NSCLC with Hsp90 inhibitors resulted in Erk dephosphorylation as well as the degradation of Akt and the EML4-ALK oncoprotein in these tumours [22-24].

Hsp90 inhibitors are also effective at inhibiting EML4-ALK-driven tumourigenesis *in vivo* in the mouse [22,23], and the treatment of three ALK+ NSCLC patients with the Hsp90 inhibitor, IPI-504, resulted in a partial response in two of the patients and stable disease in the other [25]. Importantly, Hsp90 inhibitors are effective against tumour cells expressing ALK fusion proteins that possess mutations that render them resistant to the ALK inhibitor, Crizotinib [24,26]. Thus, Hsp90 inhibitors may be useful in treating patients that develop resistance to ALK inhibitors.

One aspect of Hsp90 biology that is largely unstudied in ALK-expressing tumours is the role of Hsp90 co-chaperones. Many functions of Hsp90 are dependent on its association with co-chaperone proteins [19,20]. Co-chaperones mediate various aspects of Hsp90 function, including the association of Hsp90 with client proteins and the regulation of Hsp90 ATPase activity [19,20]. Cyclophilin 40 (Cyp40), FK506-binding protein (FKBP) 51, and FKBP52 are members of the immunophilin family of Hsp90 co-chaperones. This family is best characterized for its association with Hsp90-steroid hormone receptor complexes containing client proteins such as the glucocorticoid, estrogen, progesterone, and androgen receptors [27-30]. The individual immunophilin family members show some preference for specific hormone receptors, and they can both antagonize and promote the transcription mediated by these receptors. For example, FKBP51 inhibits the transcriptional activity of the glucocorticoid receptor [31-33], while FKBP52 is important for promoting the transcriptional activity of this receptor [32-35]. In addition to steroid hormone receptors, immunophilin co-chaperones have been found to complex with the Lck [36] and Fes [37] tyrosine kinases. As well, the expression and activity of ectopically expressed v-Src oncoprotein in *Saccharomyces cerevisiae* is dependent on the Cyp40 homolog, Cpr7 [38]. Immunophilin co-chaperones are important in cancer, as Cyp40 and FKBP51 have been shown to promote the proliferation of androgen-dependent and androgen-independent prostate cancer cell lines [39].

We identified Cyp40 in a mass spectrometry screen designed to identify proteins regulated by the JunB transcription factor in ALK+ ALCL (R.J.I and J.D.P; unpublished observation). JunB is an AP-1 family transcription factor that is highly expressed in ALK+ ALCL [40-42], and has been shown to promote the proliferation of the Karpas 299 ALK+ ALCL cell line [43]. This transcription factor also promotes the expression of CD30 [44,45] and the cytotoxic protein, Granzyme B [46], in ALK+ ALCL, which are phenotypic characteristics of this lymphoma [1,47]. Since co-chaperone proteins are important for Hsp90 function, and Hsp90 activity is critical in ALK+ ALCL, we were intrigued by our observation that JunB might promote the expression of Cyp40 in ALK+ ALCL. In this study, we examined whether the expression of the immunophilin co-chaperones was regulated by oncogenic signalling in ALK+ ALCL. We also investigated whether the immunophilin co-chaperone proteins were important for the viability of ALK+ ALCL cell lines*.* We found that NPM-ALK induced the transcription of two immunophilin family co-chaperones, *Cyp40* and *FKBP52*, but that only *Cyp40* transcription was promoted by JunB. In addition, knocking-down the expression of Cyp40, but not FKBP51 or FKBP52, reduced the viability of ALK+ ALCL cell lines. However, knock-down of the immunophilin proteins did not appear to regulate NPM-ALK stability or activation. In conclusion, we demonstrate that some members of the immunophilin family of Hsp90 co-chaperone proteins are targets of NPM-ALK signalling, and that Cyp40 plays an important role(s) in ALK+ ALCL that is not shared by other immunophilin family co-chaperones.

## Methods

### Reagents and cDNA constructs

The monoclonal antibodies (mAbs) against JunB (C-11 and 204C4a), FKBP51, FKPB52, STAT3, phospho-STAT3 (Tyr 705), Myc, and β-actin were from Santa Cruz Biotechnology (Santa Cruz, CA). The Cyp40 polyclonal antibody was also from Santa Cruz Biotechnology. The anti-JunB (C-11) mAb was used for western blotting, while the anti-JunB (204C4a) mAb was used in EMSA experiments. The anti-tubulin mAb was from Calbiochem (San Diego, CA), the anti-ALK mAb from Dako (Burlington, ON, Canada), and the anti-phosphotyrosine mAb (4G10) was from Millipore (Billerica, MA). Anti-phospho-ALK (Tyr 338, 342, and 343 of NPM-ALK) and anti-Akt antibodies were purchased from Cell Signalling Technology (Danvers. MA). Short interfering RNA (siRNA) oligonucleotides were purchased from Dharmacon RNAi Technologies (Lafayette, CO). The NPM-ALK inhibitor, Crizotinib, was generously provided by Pfizer [7,48,49]. To generate the human *Cyp40* promoter–driven luciferase reporter construct, we PCR amplified the *Cyp40* proximal promoter (−691 to +62 relative to the transcriptional start site) from the Karpas 299 cell line and cloned it into the pGL2 basic luciferase vector (Promega; Madison, WI). The AP-1 consensus sequence in the *Cyp40* promoter was mutated from TGATTCA to TAACTAA to generate the AP-1 mutant construct. The Myc-tagged JunB construct was generated by adding a double myc tag to the 5′ end of the human JunB cDNA. This was then cloned into the pcDNA 3.1A eukaryotic expression vector (Invitrogen; Burlington, ON, Canada).

### Cell lines and electroporations

The Karpas 299 and SUP-M2 ALK+ ALCL cell lines were cultured in RPMI 1640 supplemented with 10% heat-inactivated FBS, 2 mM L-glutamine, 1 mM sodium pyruvate, and 50 μM 2-mercaptoethanol. For transfections involving siRNAs, 4 × 10^6^ cells were transfected by electroporation with 100 nM pooled siRNA as previously described [50]. Cells were then incubated for 48 h at 37°C prior to analysis. For luciferase reporter assays, 1 × 10^7^ cells were transfected with 10 μg of the indicated pGL2 luciferase construct and 1 μg of a constitutively expressed *Renilla* luciferase construct (to control for transfection efficiency). In luciferase experiments involving siRNAs, cells were also transfected with 100 nM pooled control (non-targeting) or JunB siRNA. For luciferase assays performed on Karpas 299 cells over-expressing JunB, cells were transfected with the luciferase constructs as described above and 5 μg of Myc-tagged JunB or empty vector. Cells were then incubated for 24 h at 37°C prior to analysis of luciferase activity (see below).

### Cell lysis, immunoprecipitations, and western blotting

Cells were lysed in Nonidet P-40 lysis buffer [50] containing protease inhibitor cocktail (Sigma-Aldrich; Mississauga, ON, Canada), 1 mM phenylmethylsulfonylfluoride, and 1 mM sodium orthovanadate. Lysates were cleared of detergent-insoluble material by centrifugation at ~20,000 *g* for 10 min. The protein concentration of cleared lysates was determined using the BCA Protein Assay kit (Thermo Scientific; Waltham, MA). Anti-ALK immunoprecipitations were performed by incubating cleared lysates with 0.5 μg of the anti-ALK antibody and Protein A-Sepharose beads (Sigma-Aldrich) for 1–2 h at 4°C on a nutator. Beads were subsequently washed with lysis buffer and bound proteins eluted by boiling in SDS-PAGE sample buffer. Cell lysates or immunoprecipitates were resolved on SDS-PAGE gels and transferred to nitrocellulose membranes. Western blots were visualized using SuperSignal West Pico Chemiluminescent Substrate (Thermo Scientific) and band intensities quantified using a LI-COR Odyssey Infrared Imager (LI-COR Biosciences; Lincoln, NE). Expression of the quantified proteins were normalized to tubulin levels and expressed relative to control (non-targeting) siRNA-treated cells. The number of independent replicates for each experiment are indicated in the figure legends. To reprobe blots, membranes were stripped in 0.1% TBST, pH 2 prior to incubation with the new primary antibody.

### Quantitative RT-PCR (qRT-PCR)

After collection using the RNeasy mini kit (Qiagen; Mississauga, ON), total RNA was digested with DNase I to remove potential DNA contamination, and then reverse transcribed to cDNA using the Superscript II Reverse Transcriptase System (Invitrogen; Burlington, ON, Canada). qRT-PCR was performed using PerfeCTa SYBR Green FastMix (Quanta Biosciences; Gaithersburg, MD) on an Eppendorf Mastercycler realplex^4^ thermal cycler. *Cyp40* and *FKBP52* mRNA levels were then determined using the ΔΔ-CT method [51] with *β-actin* as the housekeeping gene. The following primers were used: *Cyp40* forward - TCGAGTCTTCTTTGACGTGGA, reverse - CAGTCGTGTGTCCAATGCCTT; FKBP52 forward - TGCTGAAGGTCATCAAGAGAGAG, reverse - ATGGTGGCTATGGCAATGTC; actin forward - AGAAAATCTGGCACCACACC, reverse - TAGCACAGCCTGGATAGCAA. Results are displayed relative to control siRNA-transfected cells and represent the mean and standard deviation of three independent experiments.

### Luciferase assays

Luciferase assays were performed on a BMG Labtech Plate Reader using the Dual-Glo Luciferase Assay System (Promega) and the protocol provided by the manufacturer. *Cyp40* promoter-driven firefly luciferase and constitutive *Renilla* luciferase activity were determined in triplicate for each sample. The level of firefly activity was normalized to *Renilla* activity and triplicate measurements were averaged. Three independent replicates were performed for each experiment.

### Electrophoretic mobility shift assay (EMSA)

Nuclear extracts were collected from Karpas 299 cells using the ProteoJET cytoplasmic and nuclear protein extraction kit (Fermentas; Burlington, ON, CA). EMSAs were performed with the LightShift chemiluminescent EMSA kit (Thermo Scientific) using a biotinylated probe corresponding to a 20 nucleotide sequence surrounding the AP-1 site of the *Cyp40* promoter (TTGTACTGATTCATGTCTTT). The unlabeled AP-1 mutant competitor contained the same mutation as described for the luciferase reporter construct (see above). Binding reactions were performed with 7.5 μg of nuclear protein extract, 100 fmol of the *Cyp40* promoter probe, and a 50-fold molar excess of an unlabeled *Cyp40* promoter as a competitor. For super-shift experiments, 1 μg of the indicated antibody was pre-incubated with the reaction mixture for 15 min on ice prior to addition of the biotinylated probe.

### MTS viability assays

After transfection with the indicated siRNAs, cells were resuspended to 4 × 10^4^ cells/ml and incubated at 37°C for 48 h. The number of viable cells in each sample was determined in triplicate using the CellTiter 96 AQ_ueous_ Non-Radioactive Cell Proliferation Assay (MTS assay) (Promega). Triplicate measurements were then averaged and the percentage of viable cells determined relative to cells transfected with control siRNA. Each experiment was performed in quadruplicate.

### Statistical analysis

Statistical analysis was performed using paired, one-tailed *t*-test in all cases, except the comparison of viability with Cyp40 siRNA to combined siRNA in which an unpaired, one-tailed *t*-test was performed.

## Results

### JunB promotes Cyp40, but not FKBP51 or FKBP52, expression in ALK+ ALCL cell lines

**Figure 1:**
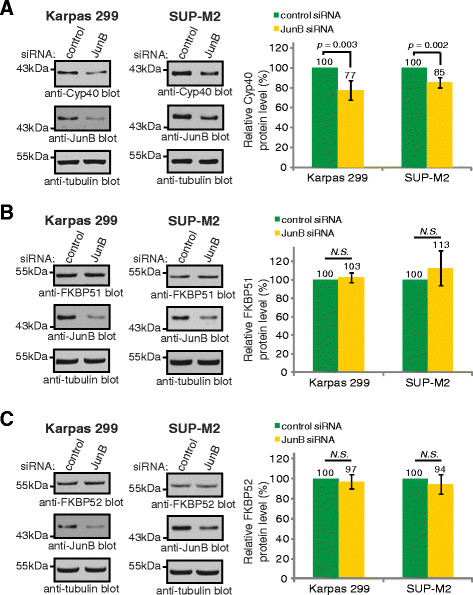
**JunB promotes Cyp40, but not FKBP51 or FKBP52, protein expression in ALK+ ALCL.** Western blot analysis (left) and quantification (right) of Cyp40 **(A)**, FKBP51 **(B)**, and FKBP52 **(C)** levels in lysates collected from Karpas 299 and SUP-M2 cells treated with pooled control (non-targeting) or JunB siRNA. Quantification represents the mean and standard deviation of five (Cyp40) or four (FKBP51 and FKBP52) independent experiments. *p* values were obtained using paired, one-tailed *t*-tests. *N.S.* indicates a *p* value >0.05.

To confirm our mass spectrometry findings showing that JunB promotes the expression of Cyp40 in ALK+ ALCL, we performed western blotting experiments. Despite incomplete JunB knock-down, we observed a decrease in Cyp40 protein expression after knock-down of JunB with siRNA in both the Karpas 299 and SUP-M2 ALK+ ALCL cell lines (Figure 1A). Since Cyp40 belongs to the immunophilin family of Hsp90 co-chaperone proteins, which includes FKBP51 and FKBP52, we also examined whether JunB promotes the expression of these proteins. However, we found that JunB knock-down did not influence FKBP51 or FKBP52 protein expression in ALK+ ALCL cell lines (Figure 1B and C).

**Figure 2:**
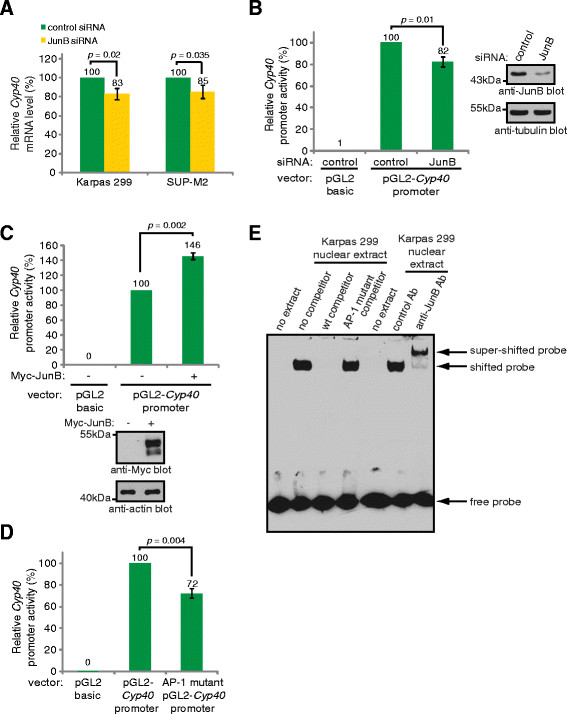
**JunB promotes*Cyp40*transcription in ALK+ ALCL. (A)** qRT-PCR analysis of *Cyp40* mRNA levels in Karpas 299 and SUP-M2 cells transfected with pooled control or JunB siRNA. **(B)** Luciferase activity in Karpas 299 cells transfected with a human *Cyp40* promoter-driven (pGL2-*Cyp40* promoter) or promoter-less (pGL2 basic) luciferase vector, and pooled control or JunB siRNA (left). Results are presented relative to the activity present in cells co-transfected with pGL2-*Cyp40* promoter and control siRNA. A western blot demonstrating the level of JunB silencing is shown (right). **(C)** Luciferase activity in Karpas 299 cells transfected with the luciferase constructs described in **(A)** along with empty vector (-) or Myc-tagged JunB (+) (top). A western blot illustrating the expression of the Myc-tagged JunB is also included (bottom). **(D)** Luciferase activity in Karpas 299 cells transfected with the pGL2 basic, pGL2-*Cyp40* promoter, or AP-1 mutant pGL2-*Cyp40* promoter luciferase construct. Luciferase activity is expressed relative to the activity present in the pGL2-*Cyp40* promoter transfected cells. **(E)** EMSAs were performed using a biotinylated *Cyp40* AP-1 probe and no competitor, an unlabeled *Cyp40* AP-1 competitor (wt competitor), an unlabeled *Cyp40* competitor with a mutation in the AP-1 site (AP-1 mutant competitor) or the anti-JunB or isotype control (control) antibody (Ab). In all experiments, error bars represent the standard deviation of three independent experiments. *p* values were determined using paired, one-tailed *t*-tests.

We next examined *Cyp40* mRNA levels after treatment of cells with JunB siRNA, and found that knock-down of JunB resulted in decreased levels of *Cyp40* mRNA in both Karpas 299 and SUP-M2 cells (Figure 2A). We also generated a luciferase reporter construct where expression of firefly *luciferase* is under control of the human *Cyp40* promoter. When transfected into Karpas 299 cells this construct exhibited strong luciferase activity, which was reduced when cells were co-transfected with JunB siRNA (Figure 2B). In addition, over-expression of Myc-tagged JunB was found to promote transcription from this luciferase promoter construct, further demonstrating that JunB promotes transcription of *Cyp40* (Figure 2C). The *Cyp40* promoter contains a consensus sequence for AP-1 family transcription factors [52] that could be recognized by JunB. Mutation of this site resulted in reduced luciferase activity (Figure 2D), demonstrating this site is important for *Cyp40* transcription. To examine whether JunB can bind this AP-1 site we performed EMSA experiments (Figure 2E). We found that a protein(s) expressed by Karpas 299 cells bound to a biotinylated probe corresponding to the AP-1 site in the *Cyp40* promoter. We further found that JunB was a major component of the probe/protein complex(es) bound to this AP-1 site, as inclusion of an anti-JunB antibody in the binding reaction resulted in an almost complete super-shift of the probe/protein complex. Taken together, our results argue that JunB functions as a direct transcriptional activator of *Cyp40* in ALK+ ALCL.

### NPM-ALK promotes Cyp40 and FKBP52, but not FKBP51, expression

**Figure 3:**
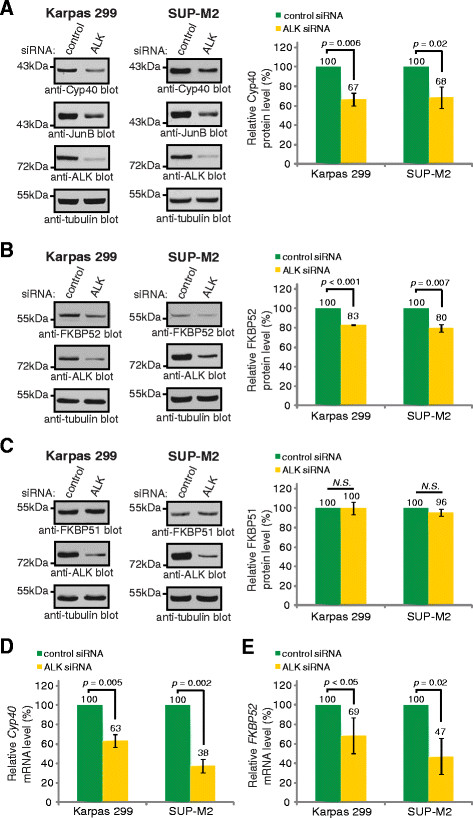
**NPM-ALK promotes Cyp40 and FKBP52, but not FKBP51, expression in ALK+ ALCL.** Western blot analysis (left) and quantification (right) of Cyp40 **(A)**, FKBP52 **(B)**, and FKBP51 **(C)** protein levels in Karpas 299 and SUP-M2 cells transfected with pooled control or ALK siRNA. qRT-PCR analysis of *Cyp40***(D)** and *FKBP52***(E)** mRNA expression in Karpas 299 and SUP-M2 cells transfected with pooled control or ALK siRNA. Error bars represent the standard deviation of the mean of three independent experiments. *p* values were obtained using paired, one-tailed *t*-tests. *N.S.* indicates a *p* value >0.05.

The NPM-ALK oncoprotein drives much of the signalling underlying the pathogenesis of ALK+ ALCL [2,3], including the elevated expression of JunB [43,44,46]. Therefore, we next examined whether NPM-ALK promotes expression of the immunophilin co-chaperones in ALK+ ALCL. We found that knock-down of NPM-ALK in Karpas 299 and SUP-M2 cells resulted in significantly reduced Cyp40 protein levels (Figure 3A). NPM-ALK knock-down also resulted in a substantial reduction in JunB levels, that was comparable to the reduction in JunB observed after JunB siRNA treatment (compare Figure 3A and Figure 1). Knock-down of NPM-ALK also resulted in decreased FKBP52 expression, but had no effect on the expression of FKBP51 (Figures 3B and C, respectively). Using quantitative RT-PCR, we found that knock-down of NPM-ALK reduced *Cyp40* (Figure 3D) and *FKBP52* (Figure 3E) mRNA expression in ALK+ ALCL cell lines. These findings show that both *Cyp40* and *FKBP52* are transcriptional targets of NPM-ALK signalling in ALK+ ALCL.

**Figure 4:**
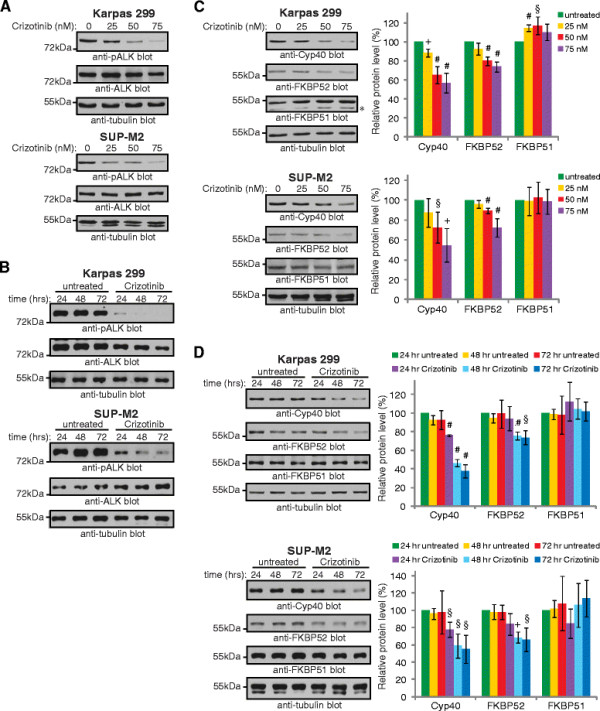
**Inhibition of NPM-ALK activity results in reduced Cyp40 and FKBP52 protein levels. (A)** Western blot analysis examining NPM-ALK phosphorylation (anti-pALK blot) in Karpas 299 (upper) or SUP-M2 (lower) cells left untreated or treated with 25, 50 or 75 nM of the ALK inhibitor, Crizotinib, for 48 h. **(B)** NPM-ALK phosphorylation in Karpas 299 (upper) or SUP-M2 (lower) cells left untreated or treated with 75 nM Crizotinib for 24, 48 or 72 h. Note: all cells were split into fresh Crizotinib-containing media 24 and 48 h post-treatment to maintain the cells in logarithmic growth. **(C & D)** Western blot analysis of Cyp40, FKBP52 and FKBP51 protein levels in Karpas 299 (upper) or SUP-M2 (lower) cells treated as in **(A)** or **(B)**. * indicates a non-specific band in the anti-FKBP51 blot. Quantification of blots are shown to the right and represent the mean and standard deviation of four **(C)** or three **(D)** independent experiments. *p* values comparing untreated cells to cells treated with each concentration of Crizotinib **(C)** or comparing treated cells at each time point to untreated cells at the 24 h time point **(D)** were obtained using paired, one-tailed *t*-tests. §*p* < 0.05, + *p* < 0.01, # *p* < 0.005.

To further examine the regulation of the immunophilin co-chaperones by NPM-ALK, we treated ALK+ ALCL cell lines with the ALK inhibitor, Crizotinib, which has been shown to be useful in treating patients with ALK+ ALCL [53] and EML4-ALK+ NSCLC [7,54,55]. Treatment of Karpas 299 and SUP-M2 cells with Crizotinib resulted in a dose- (Figure 4A) and time-dependent (Figure 4B) decrease in NPM-ALK phosphorylation on tyrosines 338, 342, and 343. These phosphorylation sites are located within the activation loop of the kinase domain, and their phosphorylation correlates with NPM-ALK activation [56,57]. Furthermore, we observed a dose- and time-dependent decrease in Cyp40 and FKBP52 protein expression in both Karpas 299 and SUP-M2 cells after Crizotinib treatment (Figure 4C and D). In contrast, Crizotinib treatment did not decrease FKBP51 expression in either cell line; however it did result in a modest, but reproducible, increase in FKBP51 expression in the Karpas 299 cells at low Crizotinib doses (Figure 4C). Thus, similar to our NPM-ALK knock-down results, treatment of ALK+ ALCL cell lines with an NPM-ALK inhibitor resulted in reduced Cyp40 and FKBP52, but not FKBP51, expression.

### Knock-down of Cyp40 reduces the viability of ALK+ ALCL cell lines

**Figure 5:**
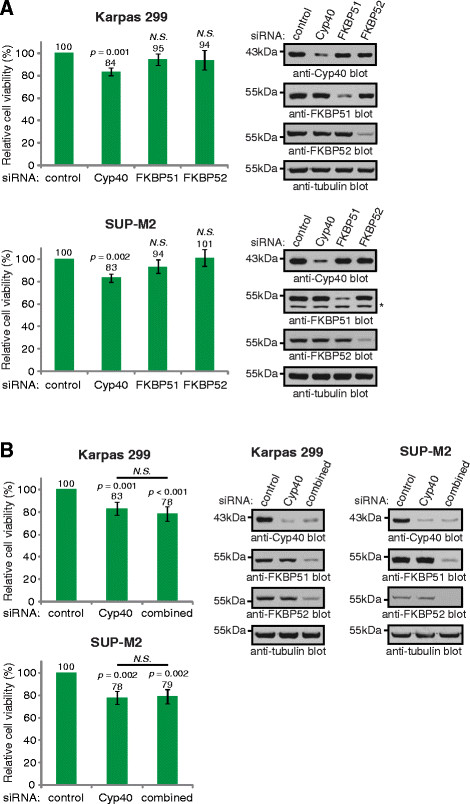
**Cyp40, but not FKBP51 or FKBP52, promotes ALK+ ALCL viability. (A)** The viability of Karpas 299 (upper) or SUP-M2 (lower) cells transfected with the indicated pooled siRNAs was measured using the MTS assay (left). Western blots (right) demonstrate the silencing efficiency of the targeted proteins. * indicates a non-specific band in the anti-FKBP51 blot. **(B)** Viability of Karpas 299 (upper left) or SUP-M2 (lower left) cells transfected with the indicated pooled siRNAs. Combined siRNA consists of siRNAs to target the three immunophilin co-chaperones, Cyp40, FKBP51 and FKBP52. Western blots (right) demonstrate the level of silencing of the indicated proteins. Quantification represents the mean and standard deviation of four independent experiments. *p* values comparing cells transfected with the indicated siRNA to those transfected with control siRNA were obtained using paired, one-tailed *t*-tests. *p* values comparing cells transfected with Cyp40 or combined siRNA were obtained using unpaired, one-tailed *t*-tests. *N.S.* indicates a *p* value >0.05.

Hsp90 is vitally important for the proliferation and survival of ALK+ ALCL cell lines [15,18], and is required for the expression and/or activation of important signalling proteins in this lymphoma [15-18,21]. Therefore, we examined whether the immunophilin co-chaperones were similarly important in ALK+ ALCL by examining the effect of their knock-down on cellular viability. Treatment of cells with Cyp40 siRNA resulted in a significant reduction in viability in both Karpas 299 and SUP-M2 cells as measured by MTS assay (Figure 5A). However, we found that reducing the expression of either FKBP51 or FKBP52 did not affect the viability of these cell lines (Figure 5A). The immunophilin co-chaperones associate with some of the same Hsp90-client protein complexes [37,58,59]; therefore, we examined whether knock-down of FKBP51 and FKBP52 in combination with Cyp40 resulted in a greater reduction in viability compared to knock-down of Cyp40 alone. Knock-down of all three immunophilin family members in combination did not significantly reduce viability over Cyp40 knock-down alone in Karpas 299 and SUP-M2 cells (Figure 5B). This finding argues that the reduced viability observed in these cell lines is predominantly due to decreased Cyp40 expression.

### Cyp40 knock-down does not affect NPM-ALK levels or tyrosine phosphorylation, nor the tyrosine phosphorylation of cellular proteins in ALK+ ALCL

**Figure 6:**
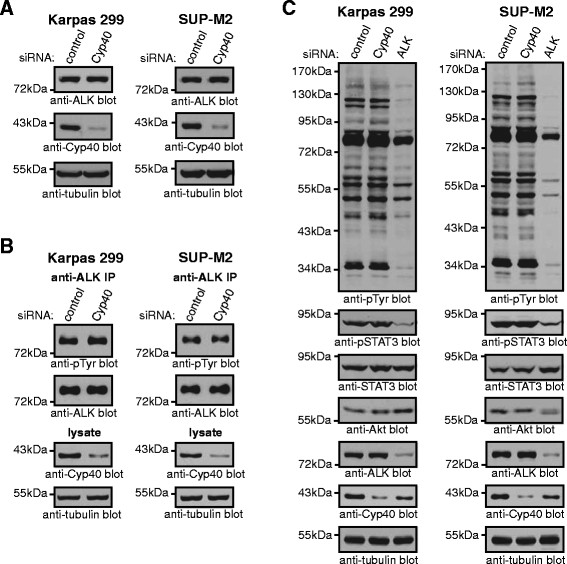
**Cyp40 does not regulate NPM-ALK expression or appear to influence NPM-ALK signalling. (A)** Western blot analysis of NPM-ALK protein levels (anti-ALK blots) in lysates collected from Karpas 299 and SUP-M2 cells after transfection with pooled control or Cyp40 siRNA. **(B)** The tyrosine phosphorylation status of NPM-ALK was analyzed by immunoprecipitation of NPM-ALK (anti-ALK IP) followed by anti-phosphotyrosine (pTyr) western blotting in cells treated as in **(A)**. **(C)** The tyrosine phosphorylation of total cellular proteins (anti-pTyr blot), the phosphorylation status of STAT3 (anti-pSTAT3 blot), and total Akt levels were analyzed by western blotting in lysates from cells transfected with pooled control, Cyp40, or ALK siRNA.

Cyp40 is primarily noted for its role in co-chaperoning with Hsp90 in complex with steroid hormone receptors [27-30]. However, Cyp40 has also been found in Hsp90/kinase client complexes. For example, Hsp90/Cyp40 complexes associate with the Lck [36] and Fes [37] tyrosine kinases, and the stability and signalling capacity of ectopically expressed v-Src in *S. cerevisiae* is dependent on the yeast Cyp40 homolog, Cpr7 [38]. Therefore, we examined whether the decrease in viability due to Cyp40 knock-down could be attributed to a failure of Cyp40 to help Hsp90 stabilize NPM-ALK and/or allow NPM-ALK to signal. We observed no difference in NPM-ALK levels (Figure 6A) or tyrosine phosphorylation (Figure 6B) in Karpas 299 and SUP-M2 cells treated with Cyp40 siRNA compared to control siRNA. Moreover, we saw no significant alteration in the tyrosine phosphorylation of total cellular proteins after Cyp40 knock-down (Figure 6C). However, knock-down of NPM-ALK in these cell lines resulted in a dramatic reduction in the tyrosine phosphorylation of cellular proteins (Figure 6C). We also observed no effect on phosphorylation of STAT3 on tyrosine 705 after knock-down of Cyp40 (Figure 6C). Phosphorylation of STAT3 on this residue is promoted by NPM-ALK signalling [60-62] and is critical for STAT3 DNA binding and transcriptional activity [63-65]. We also found no alteration in the levels of Akt (Figure 6C), which is a known Hsp90 target in this lymphoma [15]. Thus, while Cyp40 is important for the viability of ALK+ ALCL cell lines, our results argue that it does not appear to be influencing viability through regulating NPM-ALK levels or activity, or levels of the Hsp90 client protein Akt.

## Discussion

**Figure 7:**
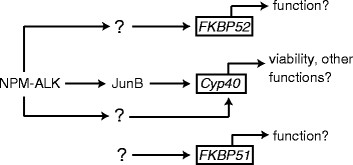
**Pathways influencing expression of the immunophilin family of co-chaperones in ALK+ ALCL.** The transcription of *Cyp40* is promoted by signals initiated by NPM-ALK, and we postulate that up-regulation of JunB by NPM-ALK accounts for much of the increase in *Cyp40* transcription. However, additional signalling pathways activated by this oncoprotein likely also contribute to *Cyp40* expression. The transcription of *FKBP52* is promoted by NPM-ALK in ALK+ ALCL, but in a manner that is independent of JunB. Although expressed in ALK+ ALCL, FKBP51 expression is not regulated by NPM-ALK or JunB in this lymphoma.

ALK+ ALCL express the three related immunophilin co-chaperones, Cyp40, FKBP51, and FKBP52; however, our findings demonstrate their expression is distinctly regulated in this lymphoma (Figure 7). Signals originating from NPM-ALK promote the expression of Cyp40 and FKBP52, but not FKBP51; whereas the only immunophilin family member regulated by JunB in ALK+ ALCL is Cyp40. Of note, we were only able to silence JunB expression by ~50% (see Figure 1), so we are likely underestimating the contribution JunB is making to *Cyp40* transcription. Since the expression of JunB is promoted by NPM-ALK in ALK+ ALCL [43,44,46], we think it is likely that NPM-ALK promotes the transcription of *Cyp40* largely through JunB. However, it is unresolved whether NPM-ALK regulates *Cyp40* transcription exclusively through JunB or via a combination of JunB-dependent and independent pathways. NPM-ALK knock-down results in a greater reduction in Cyp40 expression that JunB knock-down (compare Figures 1 and 2 to Figure 3), despite a similar reduction in JunB levels in both instances, so we believe it likely that other signalling pathways activated by NPM-ALK also contribute to Cyp40 expression. Moreover, since JunB does not influence FKBP52 expression, this demonstrates NPM-ALK signalling promotes the transcription of *FKBP52* through other downstream effectors. We were surprised by our finding that FKBP51 protein expression was modestly up-regulated in Karpas 299 cells treated with low concentrations of Crizontinib (Figure 4C). However, since we did not observe this increase in FKBP51 protein expression in Crizotinib-treated SUP-M2 cells (Figure 4C), or in Karpas 299 or SUP-M2 cells treated with ALK siRNA (Figure 3C), we are unsure of the significance of this observation.

While this is the first report to show an important role for an immunophilin co-chaperone in lymphoma, several reports have demonstrated that this family of proteins perform critical functions in other malignancies. For example, knock-down of either Cyp40 or FKBP51 in prostate cancer cell lines decreased cellular proliferation; this was particularly evident in androgen-dependent cell lines where these co-chaperones promote the transcriptional activity of the androgen receptor [39]. Metastatic melanoma has high levels of FKBP51, and knock-down of FKBP51 sensitized the SAN melanoma cell line to ionizing radiation [66]. This response was postulated to be due to decreased anti-apoptotic signalling through NF-κB in response to reduced FKBP51 levels [66]. In contrast, reducing the expression of FKBP51 in breast, lung, and pancreatic cancer cell lines resulted in reduced sensitivity to chemotherapeutic agents [67]. It was suggested in this study that activation of Akt was partially responsible for this decreased sensitivity. Thus, the immunophilin co-chaperones perform important functions in a number of cancers, and may represent attractive therapeutic targets in some malignancies.

An important unanswered question arising from our study is why reducing Cyp40 expression in ALK+ ALCL cell lines resulted in reduced viability (Figure 5). Specific experiments to determine whether this is an increase in apoptosis, a decrease in proliferation, or combination of both of these processes have been inconclusive. This decrease in viability does not appear to be due to an impairment of NPM-ALK activity (Figure 6), and suggests that the dysregulation of another protein(s) is important for this phenotype. In addition to steroid hormone receptors and kinases, Cyp40 is known to associate with a number of other proteins with a variety of cellular functions including the c-Myb transcription factor [68], mutant forms of p53 [69], and the RACK1 scaffolding protein [70]. Also, a genetic study in *Arabidopsis* identified an important role for the *Cyp40* orthologue, *SQUINT*, in microRNA biogenesis [71]. Thus, there are several cellular activities whose disruption could account for the decreased viability observed when Cyp40 is knocked down in ALK+ ALCL cell lines. Regardless of the exact cellular activity or activities regulated by Cyp40 that is important for the viability of ALK+ ALCL cell lines, our results clearly show these activities are not redundant with FKBP51 and FKBP52.

Our results show that Cyp40 does not regulate NPM-ALK levels or activity (Figure 6), but it is possible that other co-chaperones could be working with Hsp90 to regulate NPM-ALK activity. There are currently more than 20 known Hsp90 co-chaperones [19,20]. One of these proteins, Cdc37, co-chaperones for many kinase client proteins including Erb-B2, c-Raf, CDK4, CDK6 and Akt [72]. Cdc37 was identified by mass spectrometry as an NPM-ALK associated protein [73], and has also been shown to complex with EML4-ALK in NSCLC [22]. These studies however, did not examine whether these interactions are important for the activity of the respective ALK fusion proteins. We are currently investigating whether Cdc37 or other Hsp90 co-chaperones influence NPM-ALK activity. If a co-chaperone protein that cooperates with Hsp90 to regulate NPM-ALK can be identified, it could represent a potential drug target to treat ALK+ ALCL, and other cancers expressing ALK fusion proteins, especially in situations where ALK mutations have resulted in resistance to conventional ALK inhibitors.

## Conclusions

The Hsp90 chaperone protein regulates the NPM-ALK oncoprotein and other signalling molecules that promote proliferation and survival in ALK+ ALCL. Co-chaperone proteins are important co-factors of Hsp90, and in this study we examined the regulation and function of the immunophilin co-chaperones in ALK+ ALCL. We show that NPM-ALK is required for the expression of the immunophilin co-chaperones, Cyp40 and FKPB52, but not FKBP51 in ALK+ ALCL. Our findings further demonstrate that regulation of Cyp40 and FKPB52 by NPM-ALK is distinct, given that Cyp40 expression in ALK+ ALCL is promoted by the JunB transcription factor, whereas FKBP52 expression is not. Importantly, this is the first study demonstrating that signalling by an oncogenic tyrosine kinase promotes the expression of an immunophilin family co-chaperone, and identifies Cyp40 as a novel JunB transcriptional target. Finally, we demonstrate that Cyp40 promotes the viability of ALK+ ALCL cell lines in a manner that is independent of the other related immunophilin co-chaperones.

## Competing interests

The authors declare that they have no competing interests.

## Authors’ contributions

J.D.P, R.J.I, J.T.C.B, and R.L were involved in the conception and design of experiments. J.D.P performed the majority of experiments, and Z.M. performed some of the luciferase assays. J.D.P. and R.J.I drafted the original manuscript. All authors read and approved the final manuscript.

## Pre-publication history

The pre-publication history for this paper can be accessed here:

http://www.biomedcentral.com/1471-2407/12/229/prepub
